# Supplementary material for: SH2 domain protein E (SHE) and ABL signaling regulate blood vessel size
Source: bioRxiv. 2023 Jul 5:2023.07.03.547455. Preprint. [Version 2] doi: 10.1101/2023.07.03.547455 (PMC10349984; doi:10.1101/2023.07.03.547455)
Supplement: Supplement 3 [file media-3.pdf]

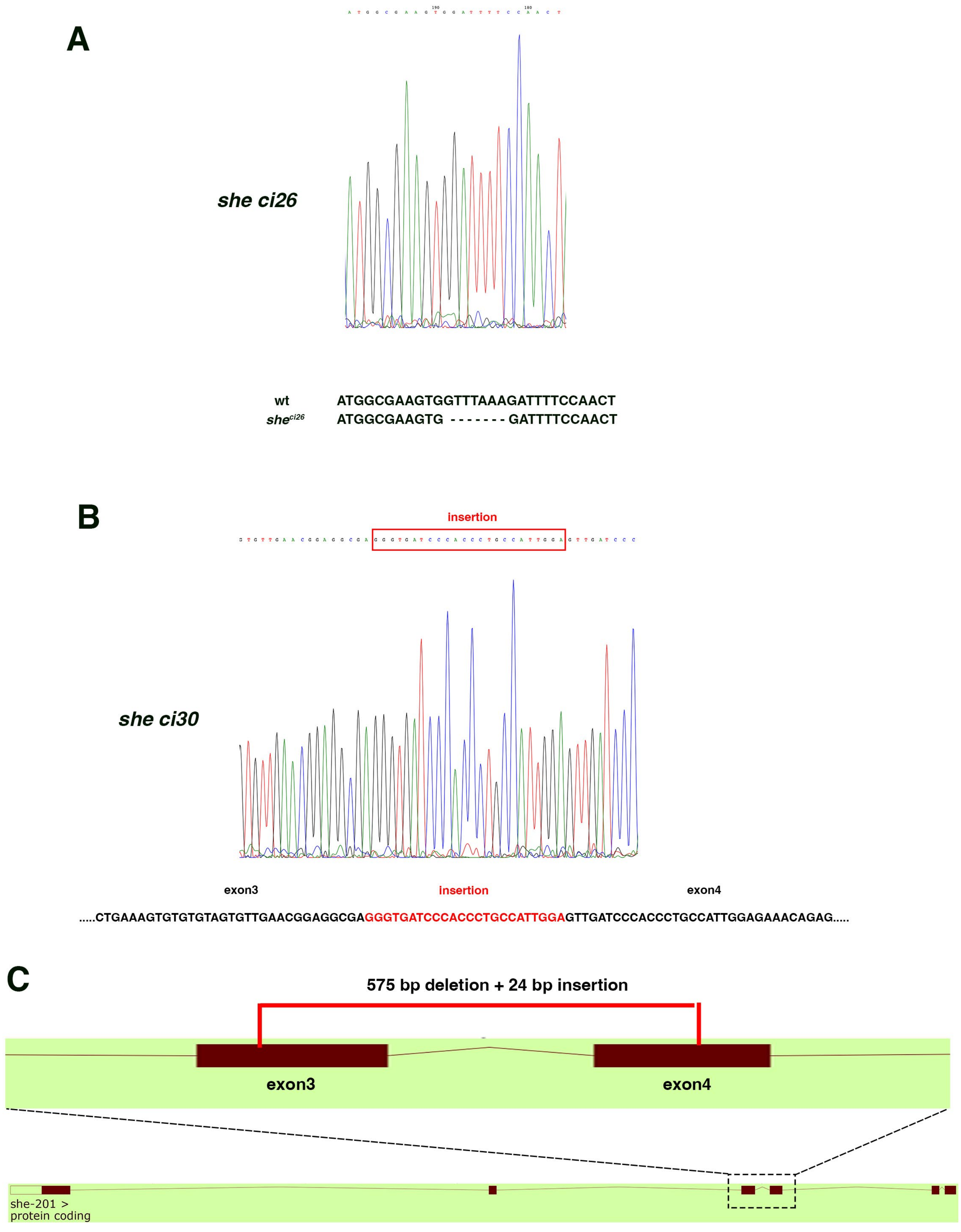

Supplementary Figure S1. Diagrams and sequence chromatograms of *she*^{ci26} and ^{ci30} mutations.

(A) *she*^{ci26} mutants have a 7 bp deletion. Alignment of wild-type and *she*^{ci26} mutant sequences is shown starting with the first coding ATG. (B) Genomic DNA sequence chromatogram of *she*^{ci30} mutants. (C) *she*^{ci30} mutants carry a 575 bp deletion and 24 bp insertion present between exons 3 and 4 within *she* gene.

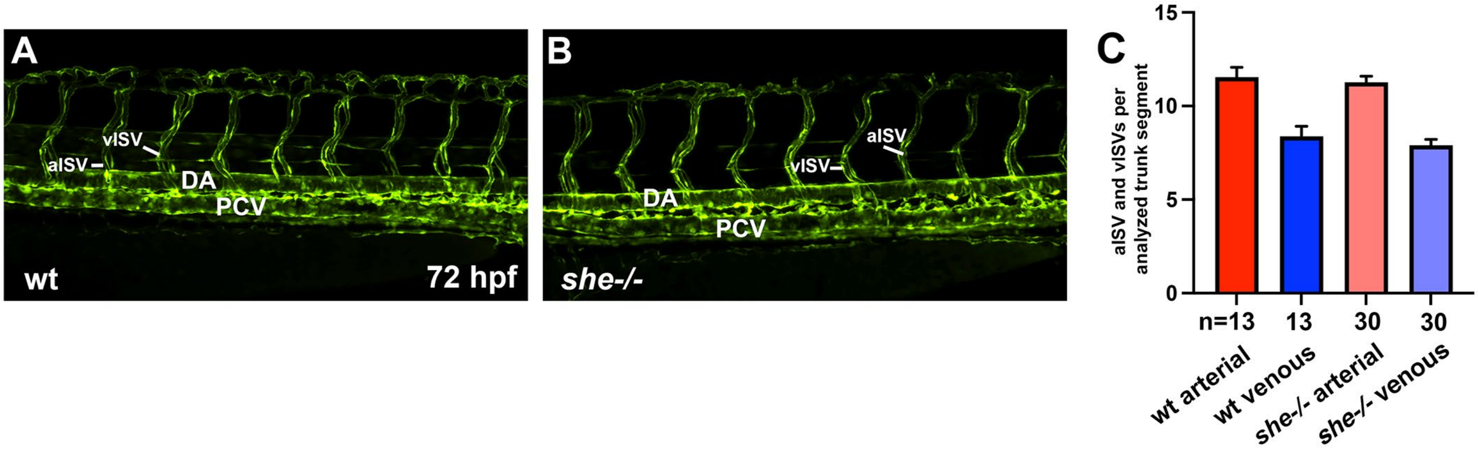

Supplemental Figure S2. Trunk intersegmental vessel analysis in *she-/-* and wt sibling embryos.
(A,B) Confocal images of the trunk region of live wt and *she-/-* sibling embryos in *kdrl:GFP* background at 72 hpf. Selected arterial (aISV) and venous (vISV) intersegmental vessels are shown. (C) The number of aISV and vISV was counted in a selected region in wt and *she* mutant embryos (the number of analyzed embryos in two independent experiments is provided as 'n'). Embryos were genotyped after the imaging. No significant difference between wt and *she* mutant embryos was observed (p>0.05, t-Student's test).

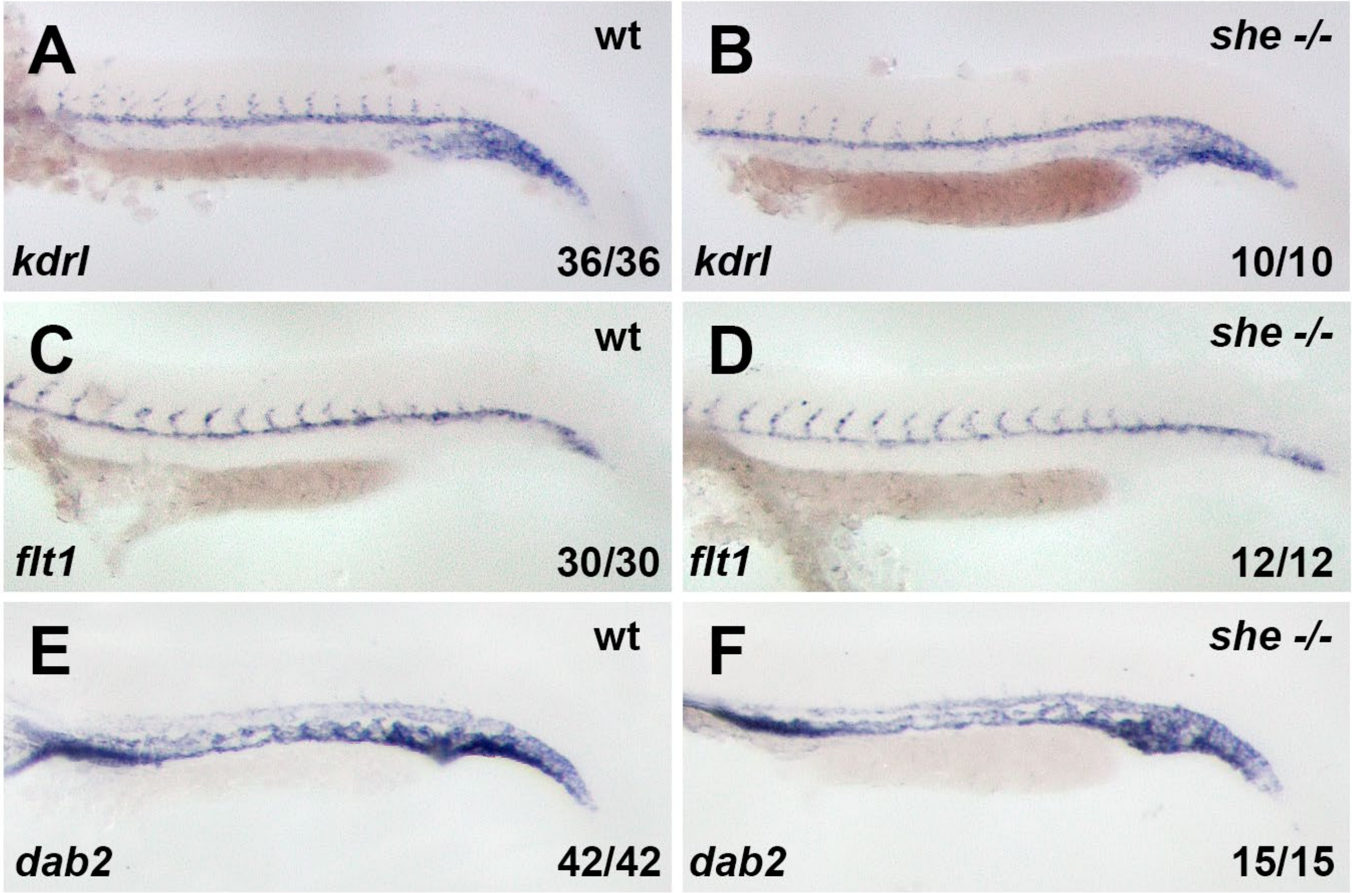

Supplemental Figure S3. Endothelial marker expression, analyzed by in situ hybridization, is unaffected in *she* mutants at 24 hpf.

(A, B) *kdrl* is strongly expressed in the dorsal aorta (DA) and intersegmental vessels (ISVs) and weakly expressed in the posterior cardinal vein (PCV) of both wild-type sibling and *she -/-* mutant embryos.

(C, D) *flt1* expression is restricted to the DA and ISVs of both wild-type and *she -/-* mutants.

(E, F) *dab2* is strongly expressed in the PCV and lightly expressed in the DA of both wild-type and *she -/-* mutants. Trunk region is shown in all panels. Embryos were obtained from an incross of *she+/-; kdrl:GFP* parents and subsequently genotyped.

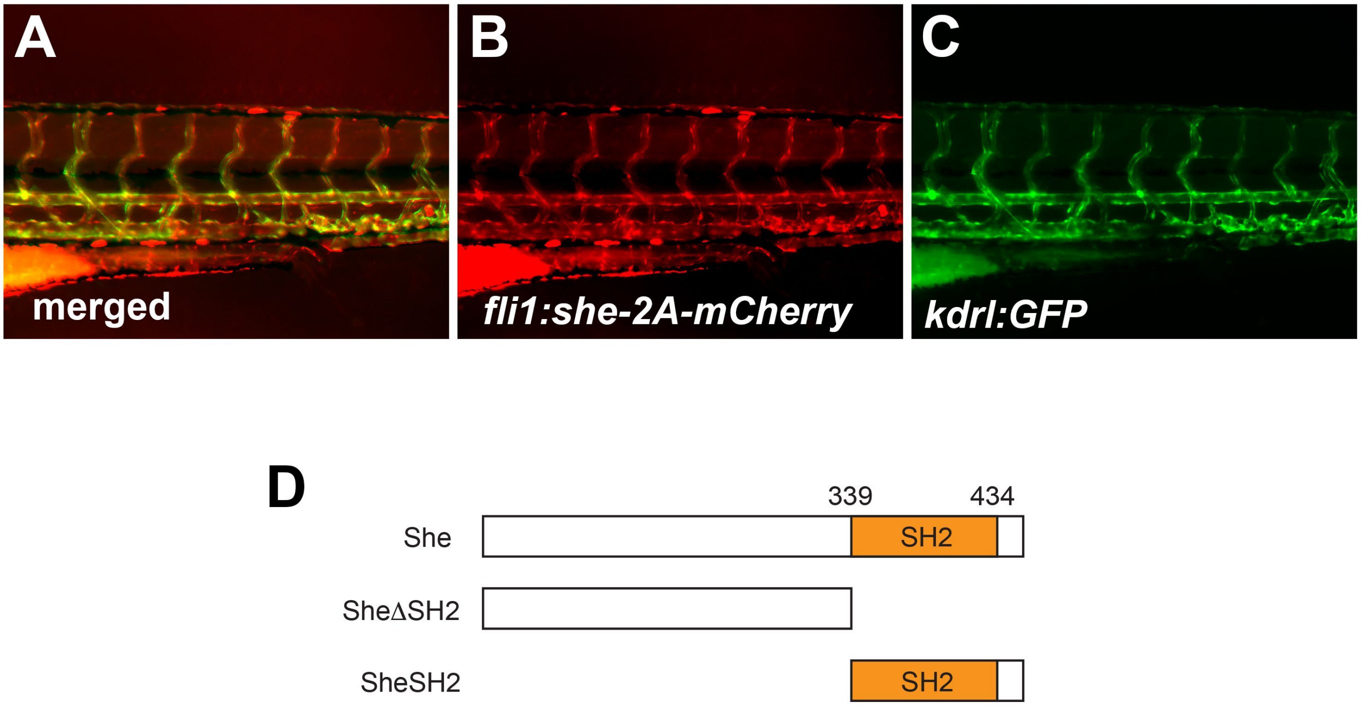

Supplemental Figure S4. Generation of wild-type and mutant vascular endothelial-specific *she* zebrafish lines. (A-C) Fluorescent microscopy image of *fli1:she-2A-mCherry; kdrl:GFP* embryo at 3 dpf. (D) A diagram of She deletion constructs.

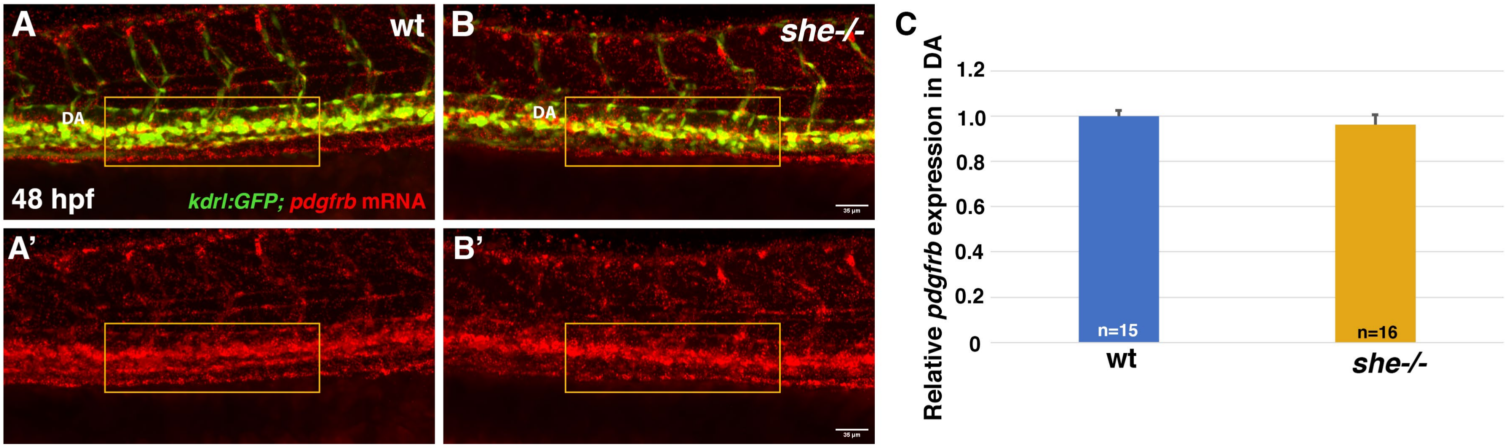

Supplemental Figure S5. *pdfgrb* mRNA expression analysis by hybridization chain reaction (HCR) at 48 hpf. (A,B) *pdgfrb* (red) expression in the trunk region of wild-type sibling and *she* mutant embryos in *kdrl:GFP* background at 48 hpf. Fluorescence within the dorsal aorta region (boxed) was selected for quantification. (C) Quantification of relative *pdgfrb* mRNA expression. No significant difference was observed (p>0.05, Student's t-test).

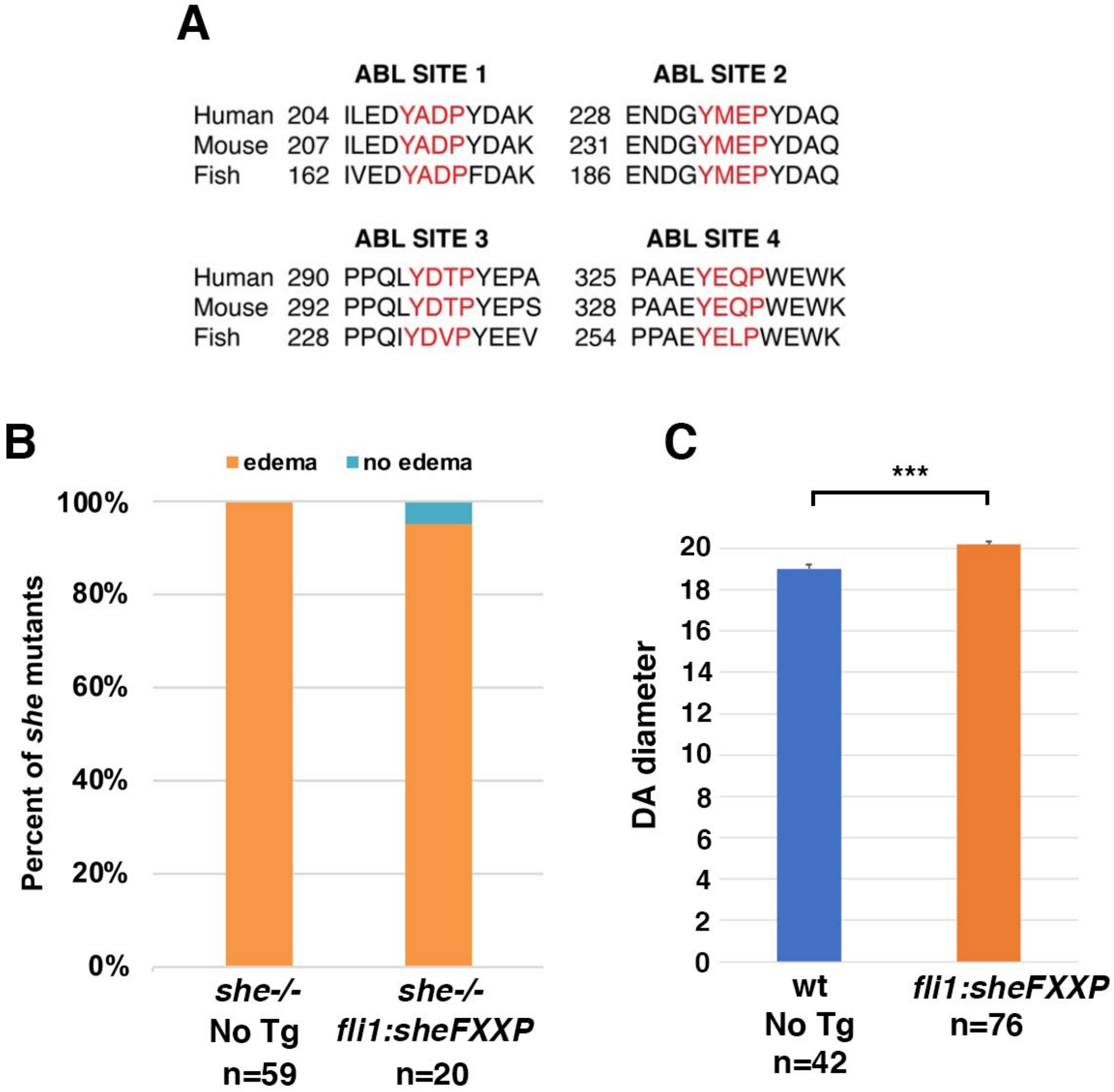

Supplemental Figure S6. Consensus ABL phosphorylation sites YXXP are required for SHE function.

(A) Consensus ABL phosphorylation sites are conserved between zebrafish, mouse and humans.

(B) Vascular endothelial expression of a mutant construct *fli1:sheFXXP-2A-mCherry,* where all four consensus tyrosines have been substituted into phenylalanine, fails to rescue the pericardial edema in she mutants at 4 dpf. The first bar showing *she-/-* embryos (no Tg) is copied from Fig. 3J.

(C) DA diameter measurement in *fli1:sheFXXP-2A-mCherry* embryos and sibling mCherry-negative embryos at 28 hpf. 5 measurements were performed in each embryo, which were then averaged for statistical calculations. n corresponds to the number of embryos. ± SEM is shown. ***$p<0.001$, Student’s t-test.

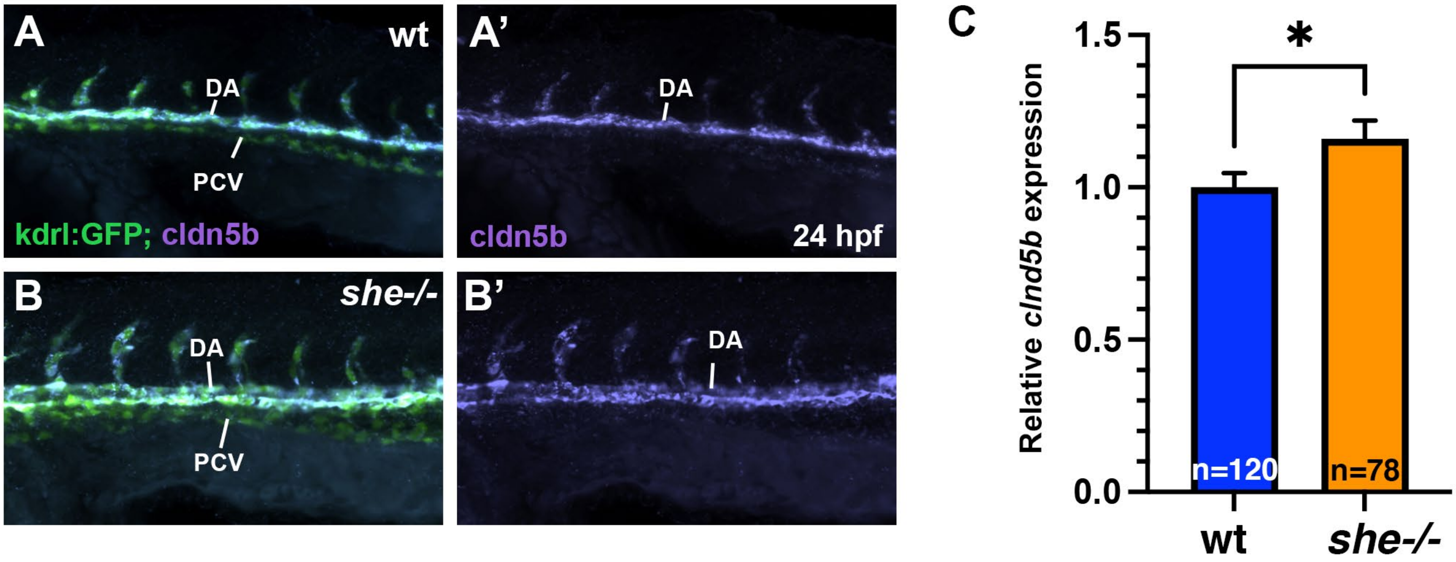

Supplemental Figure S7. Hybridization chain reaction (HCR) analysis of *cldn5b* mRNA expression at 24 hpf.
(A,B) *cldn5b* (purple) and *kdrl:GFP* fluorescence in the trunk region of *she* mutant and wild-type sibling embryos. DA, dorsal aorta; PCV, posterior cardinal vein. *cldn5b* fluorescence is shown in A',B'.
(C) Quantification of *cldn5b* fluorescence in the DA. *p=0.036, Student's t-test. Error bars show SEM. Data show combined results from two independent experiments.

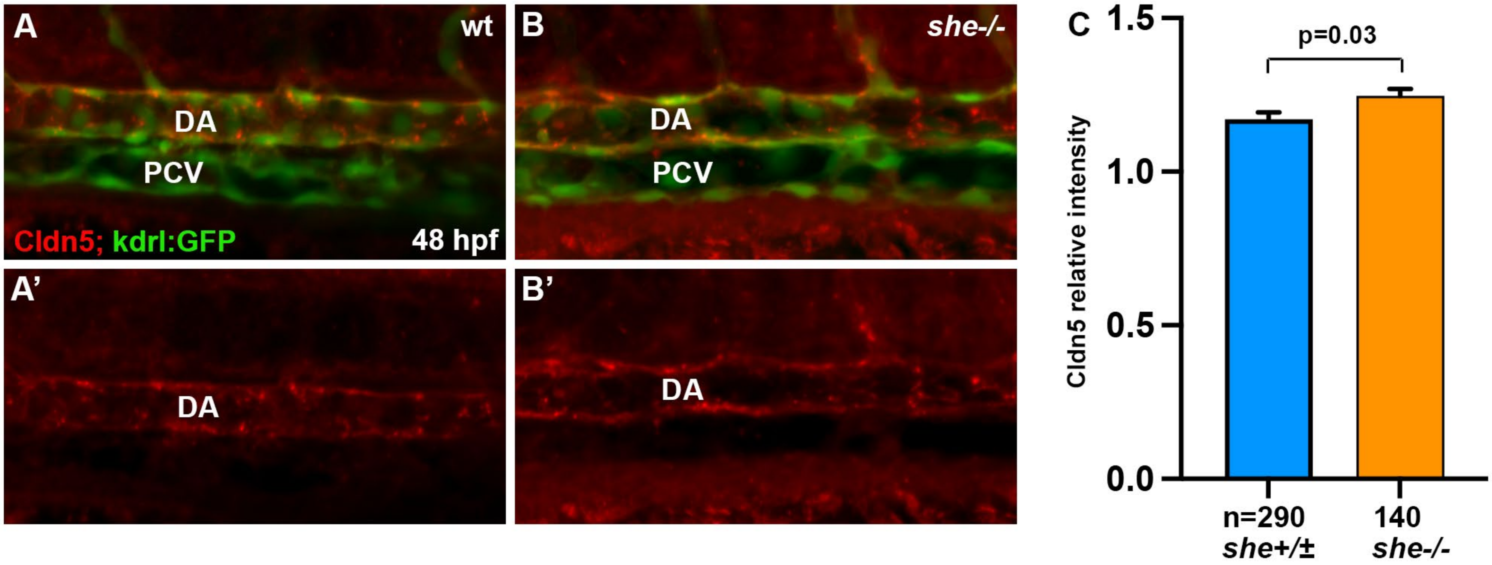

Supplemental Figure S8. Cldn5 protein expression is increased in *she* mutant embryos.
(A,B) Immunostaining for Cldn5 (red) combined with *kdrl:GFP* fluorescence in wild-type sibling and *she* mutant embryos at 48 hpf. Red channel is shown in A',B'. DA, dorsal aorta; PCV, posterior cardinal vein.
(C) Quantification of Cldn5 immunofluorescence within the DA. Embryos were obtained from an incross of *she+/-; kdrl:GFP* parents and subsequently genotyped. *she+/+* and *she+/-* genotypes were combined for this analysis. Error bars show SEM; Student's t-test was used.

Supplemental Table S1. Primer sequences used to generate she mutant and transgenic lines and perform qPCR

she_F1	ATCAATAATAGGGGCCACTTTGAG
she_R1	CCGTCCTAACTCGATGGCAG
she_gRNA1	GCGTAATACGACTCACTATAGGAGGCGAAGGTGAGGGGTCGTTTTAGAGCTAGAAATAG
she_gRNA2	GCGTAATACGACTCACTATAGGCAGGGTGGGATCAACAATGTTTTAGAGCTAGAAATAGC
she_gRNA_R1	AAAGCACCGACTCGGTGCCA
she_gRNA left	TATGCTCTTGTGCAGAGATTCG
she_gRNA right	TGACCAAAACATACCCTTTTCA
she_gRNA midR	CTGACAGGGCTCTAACAATCTG
she_attB_F1	GGGGACAAGTTTGTACAAAAAAGCAGGCTTCACCATGGCGAAGTGGTTTAAAGATTTTCC
she_attB_F3	GGGGACCACTTTGTACAAGAAAGCTGGGTTGTGTGTGCGTGGCACGGGGTG
she_deltaSH2_attB_R	GGGGACCACTTTGTACAAGAAAGCTGGGTTGCTCTGTTTCTCCAATGGCAGG
sheSH2_attB_F	GGGGACAAGTTTGTACAAAAAAGCAGGCTTCACCATGTGGTATCATGGCAGTGTGAGTCG
she_attB_R3	GGGGACCACTTTGTACAAGAAAGCTGGGTTGTGTGTGCGTGGCACGGGGTG
sheYXXP1_F1	CAGAACCGATCATCGTAGAGGATTTTGCCGACCCATTTGATGCTGAG
sheYXXP1_R1	CTCAGCATCAAATGGGTCGGCAAAATCCTCTACGATGATCGGTTCTG
sheYXXP2_F1	GAAGGAGAGAACGATGGCTTCATGGAGCCTTATGATGCCCAGCTC
sheYXXP2_R1	GAGCTGGGCATCATAAGGCTCCATGAAGCCATCGTTCTCTCCTTC
sheYXXP3_F2	GTCTGGACCCCCTCAGATCTTTGATGTCCCATATGAGGAGG
sheYXXP3_R2	CCTCCTCATATGGGACATCAAAGATCTGAGGGGGTCCAGAC
sheYXXP4_F1	CAGACCGCCGGCAGAGTTTGAGCTGCCCTGGGAGTGGAAGAAAG
sheYXXP4_R1	CTTTCTTCCACTCCCAGGGCAGCTCAAACTCTGCCGGCGGTCTG
cldn5a-F	GACAACGTGAAAGCGCGGG
cldn5a-R	AGGAGCAGCAGAGTATGCTTCCC
EF1a-F	TCACCCTGGGAGTGAAACAGC
EF1a-R	ACTTGCAGGCGATGTGAGCAG

MOVIE LEGENDS

Movie 1. Blood flow in the tail region of wild-type zebrafish embryos. Wild-type siblings obtained from an incross of *she*^{ci26} heterozygous adults in *kdrl:GFP; gata1:dsRed* background were imaged at 4 dpf using confocal microscopy.

Movie 2. Blood flow in the tail region of *she* mutant embryos. Mutant embryos obtained from an incross of *she*^{ci26} heterozygous adults in *kdrl:GFP; gata1:dsRed* background were imaged at 4 dpf using confocal microscopy.
